# Stem cell-based models of embryos: The need for improved naming conventions

**DOI:** 10.1016/j.stemcr.2021.02.018

**Published:** 2021-03-25

**Authors:** Kirstin R.W. Matthews, Daniel S. Wagner, Aryeh Warmflash

**Affiliations:** 1 Baker Institute for Public Policy-Center for Health and Biosciences, Rice University, Houston, TX 77005, USA; 2 Department of BioSciences, Rice University, Houston, TX 77005, USA

## Abstract

Stem cell-based models of embryos are known by various names, with different naming conventions, leading to confusion regarding their composition and potential. We propose the need for a general term for the field to promote public engagement and the development of a systematic nomenclature system to differentiate between specific models.

## Introduction

In 2014, scientists developed methods to culture mammalian embryonic stem cells (ESCs) to obtain predictable patterning of cell fates similar to a developing embryo at gastrulation stage (van den Brink et al., 2014; Warmflash et al., 2014). In the years since, additional research groups have created numerous novel entities mimicking various stages of early embryo development (Baillie-Benson et al., 2020; Shahbazi et al., 2019; Simunovic and Brivanlou 2017). These entities can be created from ESCs, induced pluripotent stem cells (iPSCs), and other pluripotent stem cells (PSCs) (Shahbazi et al., 2019). They offer scientists a unique tool to understand early embryo development. In this article, we refer to these entities as embryoids (for reasons described below); however, alternative names could also be used, such as stem cell-based models of embryos (SCMEs).

Embryoids have strong advantages that promote their use in research, especially for early human development. Scientists can produce embryoids in larger numbers to allow for statistical analysis, which they cannot do with embryos created via fertilization due to limited availability, funding restrictions, and ethical concerns associated with human embryos (Hyun et al., 2020). Embryoids allow for the testing and refinement of theories and hypotheses before or in place of moving on to experimenting on embryos. Embryoids can also be manipulated or generated in diverse ways to improve the experimental design. For example, colony geometries, substrate stiffness, or media composition can be varied and the associated phenotypes determined with high resolution. In addition, embryoids can be more easily genetically manipulated, allowing scientists to determine if specific genes are required by inducing mutations in the stem cells used to create them or by generating fluorescent reporter constructs that allow detailed observations of cellular events in real time.

While embryoid research does not involve using embryos, it still invokes ethical questions and challenges (Aach et al., 2017; Hyun et al., 2020). Some embryoids are a product of human ESCs; others are from non-human animals or are developed from iPSCs derived from adult cells, which do not involve the use of a human embryo, which could be viewed as avoiding some ethical concerns. Furthermore, while current human embryoid models do not exactly mimic an embryo, as scientists improve *in vitro* culture methods, some types of embryoids may approach sufficient complexity to raise concerns about the ethics of creating and using them for research. This makes it important to be able to easily differentiate between models to ensure regulators also view them separately. Another problem in the near future will be determining characteristics and/or features that could raise ethical concerns and therefore cause them to be subject to regulation similar to, but possibly less stringent than, a human embryo or fetus, which in US federal policy (45 C.F.R. §46) is defined as starting at implantation.

In addition to ethical challenges, human embryoids create a series of policy challenges related to confusion about how embryoids are created, what they are composed of, what they can do, and how they should be regulated. This is especially important in the United States, where federal funding is permitted for human ESC research but not human embryo research (Hyun et al., 2020; Matthews and Morali 2020). Furthermore, the 14-day limit on human embryo research cannot be applied to embryoids, as they can be produced to mimic different later developmental stages beyond 14 days of embryonic development, but before 14 days of culture without transitioning through the primitive streak stage (Aach et al., 2017; Hyun et al., 2020). Of course, trying to apply embryo laws and guidelines to embryoids hangs on the assumption that we should treat embryoids similar to an embryo or a cell model, when perhaps they should be considered as something else that is unique.

The complex origins and diverse applications of these entities make for potentially complicated ethical and policy questions about their use. Public concerns are exacerbated by the terms used to describe these entities. While many scientists are eager to use terms that highlight their similarity with embryos, these terms can raise unnecessary concern that the entities are embryos in the minds of the public and regulators. Names can hold power in public perception, and much of the confusion of how to regulate human embryoids is linked to contradictory and sometimes inaccurate names scientists and science journalists have used. Just like how the term “cloning” elicits concern (bringing to mind pictures of newly created identical humans), so do many of the general terms, such as “artificial embryos” used by the media. However, more specific terms easily become obtuse and jargon laden, prohibiting non-experts and new researchers from understanding the field.

These problems with names highlight the need for an agreed-upon general term that will allow the public to understand the research. In addition, scientists should develop a nomenclature structure to adequately communicate similarities and differences between cell models for scientific audiences. Ultimately, if scientists want to gain the public's trust and avoid giving rise to unnecessary regulations on human embryoids, especially for versions that are clearly not capable of developing into a fetus, then they must work to communicate to the public what these models are and are not.

## What's in a Name?

An embryo derives from fertilization of an egg cell by a sperm cell. It develops through interpretation of internal cues, patterning events, and interaction with maternal tissues to result in an entity with a three-dimensional (3D) pattern that is generally reproducible from embryo to embryo. Embryos proceed through defined stages with characteristic morphologies, such as blastula, gastrula, and neurula stages. In contrast, an embryoid is an artificial construct built from cultured cells with or without a supplied matrix or support that attempts to mimic all or part of an embryo. Embryoids may be created to mimic a specific stage without clear hallmarks of preceding stages through manipulation of the culture conditions or component cells. Scientists and the scientific media use many different names for embryoids, each with its own rationale and implications (Shahbazi et al., 2019; Simunovic and Brivanlou 2017). Early studies typically used a complex name, such as “micropatterned human ESC colonies” (Warmflash et al., 2014). Another study proposed the term “gastruloids” for a 3D model of gastrulation (van den Brink et al., 2014). The term was later expanded to include all organized models of gastrulation, including two-dimensional (2D) versions (Simunovic and Brivanlou, 2017). This more inclusive definition serves to indicate that all of these models emulate some aspect of the gastrulating embryo, and links by the “oid” suffix to additional organoid research also using PSCs (Purnell and Lavine 2019; Simunovic and Brivanlou 2017). With subsequent publications in this area, additional names were proposed for these stem cell models for early development. Surveying publications in the field since 2014, we found three broad categories of names: (1) general names used to describe all research in the area; (2) names linking the specific developmental time points being modeled; and (3) names noting cells used within the model and their characteristics (see Table 1).

**Table 1:** Examples of Names for Embryoids and Their Definitions

Name	Definitions	Citations
**General name**		

Artificial embryo	differentiating PSCs that model/mimic an embryo, most commonly used in the media and ISSCR	Wysocka and Rossant (2019)
Embryoid	first designated in 1902 to describe a tumor; more currently it refers to an organized embryoid body, made up of differentiating PSCs that model/mimic an embryo	Simunovic and Brivanlou (2017)
Embryonic organoid	differentiating PSCs that model/mimic an embryo	Turner et al. (2017)
SHEEFs	synthetic entities with embryo-like features	Aach et al., (2017)
Synthetic embryo	differentiating PSCs that model/mimic an embryo, most commonly used in the media	Denker (2014); Warmflash (2017).

**Developmental time-based name**		

Blastoid	an embryoid modeling the blastocyst stage of development	Rivron et al. (2018)
Gastruloid	2D or 3D embryoids modeling the gastrulation stage of development	van den Brink et al., (2014)
PASE	Post-implantation amniotic sac embryoid	Shao et al. (2017)

**Cell-based name**		

Micropatterned hESC colonies	2D models of the gastrulation stage of development	Warmflash et al. (2014)
ETS embryo	embryoids from embryonic and trophoblast stem cells with a 3D structure	Harrison et al. (2017)
ETX embryo	Embryoids from embryonic, trophoblast, and extra-embryonic endoderm stem cells	Sozen et al. (2018)

h, human; ISSCR: International Society for Stem Cell Research; PSCs: pluripotent stem cells.

Several general names have been used to describe the field since it emerged (Table 1). The term “embryoid” dates back to the early 1900s to describe a teratoma that contained cells of embryonic origin (Neely 1938). More recently, embryoid has been repurposed to describe these organized stem cell models (Simunovic and Brivanlou 2017). Scientists have also used terms that point to the non-natural nature of the entities, including “synthetic embryos,” “SHEEFs” (synthetic human entities with embryo-like features) and artificial embryos. Artificial embryo and synthetic embryo are used frequently by the press to describe research, often linking the work to controversies over whether it is an embryo and whether, in the United States, it should be federally funded (Regalado 2019).

Names have also been developed based on the developmental event being modeled, such as gastruloid, “blastoid,” or “PASE” (post-implantation amniotic sac embryoid) (Table 1). For example, gastruloid describes models mimicking approximately the third week of development in humans: gastrulation. This raises the question of how to define gastrulation in order to define which stem cell models are mimicking this stage. Gastrulation is a complex process involving differentiating to three germs in a spatial pattern, establishment of the primary body axes, and elongation along the anterior-posterior axis. In many organisms, a site of gastrulation is established, such as the primitive streak in mammals or blastopore lip in amphibians, and particular cellular behaviors are induced at this site. Here, we take the view that any spatially organized stem cell model that mimics any of these aspects should be referred to as a gastruloid. Therefore, the term applies to both 2D and 3D models as well as different techniques used to induce differentiation that may affect the composition of the resulting cell population, although some argue the term should be reserved for 3D models (Baillie-Benson et al., 2020; Simunovic and Brivanlou 2017). An alternative is the nomenclature system described below, which distinguishes these entities while referring to all of them with the general term gastruloids.

Some scientists developed more technical and jargon-laden terms that try to accurately describe specific entities created (Table 1). Warmflash et al. (2014) described the 2D entities as “micropatterned human ESC colonies” without referring to the germ layer patterning that occurs in these colonies. Later publications used acronyms listing types of cells used. For example, “ETX embryos” describe cells used: embryonic, trophoblast, and extra-embryonic endoderm stem cells (Sozen et al., 2018). Furthermore, some researchers try to avoid using a general name altogether. In the 2019 publication by Zheng et al. (2019), researchers refer to their embryoids as a “controllable model system to recapitulate developmental events reflecting epiblast and amniotic ectoderm development in the post-implantation human embryo.” However, subsequent news articles related to the paper refer to it as a synthetic embryo (Regalado 2019).

## The Need for a General Name and a Nomenclature System

Having a simple, unified term for diverse entities is important. It provides a clear shorthand both inside the research community and for the public. While it is appealing to coin specific terms that reflect the intention and novelty of the entities created by a specific method, it diffuses the relationships among entities. Unfortunately, broad simple terms also risk confusion. Therefore, we propose a dual naming system, one encompassing term and a set of specific terms that will provide clarity of the relationships and differences among entities.

While scientists may be content with more specific and technical terminologies to describe embryoids, this has proved to be problematic for public understanding and ultimately regulation of scientific activities. Embryoids continue to receive media coverage and captivate the public's attention because of their novelty and potential ethical questions. Technical language only creates a situation where journalists and non-experts translate the research and its implication to the public. This leads to significant misunderstanding in the public and exaggerated claims by the media of what these stem cell models are currently capable of and the ethical challenges they pose. Therefore, we suggest using one term be used to describe the entire field.

We suggest two possible terms—embryoid or SCME—that could be used, although we acknowledge further discussion is needed within the scientific community to ensure the term is fully adopted. Regardless of which name is chosen by scientists, the name should avoid using synthetic or artificial within the name, which imply the entities were created from scratch and function like a full embryo with the ability to grow to a human. These terms are problematic because the public may view the field as creating babies in a test tube. Currently, none of the models can form a functioning human embryo as most efforts are missing vital components or cell types required to develop fully, such as extra-embryonic tissues. Even if they did have these components, they still lack sufficient organization to form a full embryo.

While the broad term embryoid risks being too similar to embryo, it serves as a simple phrase suitably distinct to avoid confusion. In particular, its simplicity will increase its use and broad public acceptance. The suffix oid is widely used to refer to similarity without identity among the general public. For example, the colloquial use of humanoid is used to refer things that are human shaped but are not humans. It also resonates with the term organoid, which is widely used to indicate entities that have some features of the organ they are designed to mimic but are clearly not identical.

We propose that the embryoid designation serves to refer to models that attempt to recapitulate broad aspects of early development, such as formation of the blastula or gastrula. Above, we argued for an inclusive definition of the term gastruloid to include models that mimic any part of the gastrulation process. Here we similarly suggest this general term for models that are intended to mimic any aspect of embryonic development, with the caveat that the phenomenon they are modeling is a property of embryos rather than of a specific organ (and therefore the object would not be described as an organoid).

We acknowledge that initial confusion may arise between the historical use of the term “embryoid bodies,” which referred to a specific class of entities, and broader use of embryoids to refer to a diverse set of entities. The similarity between these terms serves to underscore the breadth of usage of embryoid, since embryoid bodies have a long history of modeling early embryogenesis, albeit more crudely than the more refined applications being applied currently.

Alternatively, we propose the use of an acronym, such as SCME. This acronym is more technical than embryoid, but still serves as a broad term that captures features of embryogenesis without requiring total identity. Adoption of more abstract terms like this has the benefits of removing preconceived notions from the minds of the public.

The issues scientists currently face in relation to public misconceptions of embryoid research is similar to past discussions around cloning. In the early 2000s, scientists wanted to distinguish between different uses of somatic cell nuclear transfer (SCNT), using the terms reproductive cloning (cloning a human being) versus therapeutic cloning (cloning human cells). However, the public continued to confuse the two areas until scientists went back to describing the technique in more technical terms as SCNT (O'Mathuna 2002). Afterward, the public discussions became less focused on cloning a human and more on the cell culture work and its potential. Using a term, such as embryoids or SCME, helps highlight the limitations of the research more effectively and point to the differences as well as similarities these entities have to natural embryos. This is quite important for public engagement, especially with policymakers wary of public backlash to human embryo research.

## Developing Specific Terminology

Overlapping terminology and unclear technical naming schemes make it challenging for scientists to communicate with the other researchers both inside and outside of the field. It also complicates discussion about regulations. Some models are confined to 2D or lack all cells/tissues necessary to develop into a complete organism. Others are more complex, and, with refinement, may eventually come closer to the potential of an embryo. If regulators cannot easily differentiate between these two categories, they could potentially limit all embryoid research in an effort to provide oversight over the latter.

Names of embryoids developed continue to evolve to include novel models or extensions of existing ones. Names with excessive jargon exclude non-experts from following the research, perhaps intentionally since it is a politically and ethically sensitive area. However, not only do they exclude public engagement, they also preclude those interested in entering the field—such as students—from accessing the literature. Scientists are also struggling with defining specific entities developed in the laboratory to communicate to other scientists, which can even delay manuscript publications (Zernicka-Goetz and Highfield, 2020). A structured nomenclature system can help scientists clarify what they are developing as well as remove barriers for understanding the field.

A nomenclature system would ideally identify the model to allow for similarities and differences with other models to be implicitly understood by the scientific community as well as regulators. Table 2 describes one nomenclature system that could address the issues of naming specific embryoids with five specific areas: cell culture dimension (2D versus 3D), animal, input cell type, modifier, and developmental stage. Within these areas, the primary description would refer to the developmental stage being modeled, and include additional modifiers to distinguish between different models for the same developmental stage. For clarity, we suggest that these additional modifiers should identify the animal of origin and the cell culture (2D versus 3D) used. In addition, the cell type should be stated to allow the reader and regulator to understand the embryoid's potential for development. The name should also describe the developmental stage or tissues the embryoid is mimicking. Finally, a modifier could be included to permit differentiating models that have subtle differences, such as noting the process or pathway by which a model was developed if different from other existing models.

**Table 2:** Proposed Nomenclature System for Embryoids

Topic	Definition	Example
Cell culture	2D versus 3D	2D
Animal	species of cell used	H
Cell type	cells used	E
Modifier	additional notes, such as process or pathway used	BMP (BMP-induced differentiation)
Model	Tissue or embryonic stage	Gastruloid

A nomenclature system should include various areas (noted here as topics) to help differentiate different embryoid models from others and note subtle but significant differences.

E, ESC.

Ultimately, the name might end up as an acronym (because of the wordiness of the nomenclature); however, it should adequately describe the entity. For example, the full name for the embryoid created by Warmflash et al. (2014) would be identified as a 2D-hE-BMP-gastruloid, which reflects that it is a 2D object, derived from human ESCs, and created by treatment with BMP (to distinguish from similar but distinct 2D-hE-gastruloids that are created with Wnt). To remove complexity, it can be abbreviated or shortened to 2D-hE-g or just gastruloid after it is first introduced using the more technical name (see Table 3 for additional suggested examples of names for recently developed embryoids).

**Table 3:** Examples of Original and New Embryoid Names Using Proposed Nomenclature System

Object	Proposed Name	References
hESC gastrulation micropatterns (BMP)	2D-hE-BMP-gastruloid	Chhabra et al., 2019; Etoc et al. (2016); Heemskerk et al., 2019; Tewary et al., 2017; Warmflash et al. (2014)
hESC gastrulation micropattern (Wnt)	2D-hE-Wnt-gastruloid	Martyn et al., 2018; Martyn et al. (2019a); Martyn et al. (2019b)
Blastoid (from mE + mTSC)	3D-mET-blastoid	Rivron et al. (2018)
Blastoid (from mE + mTSCs + XEN)	3D-mETX-blastoid	Vrij et al. (2019)
Blastoid (from mEPS cells)	3D-mEP-blastoid	Li et al. (2019)
Blastoid (from mEPS + mTS cells)	3D-mEPT-blastoid	Sozen et al., 2019
ETS embryo, ETS embryo	3D-mET-gastruloid	Harrison et al., 2017
ET/X embryo	3D-mETX-gastruloid	Sozen et al. (2018)
Pluripotent stem cell-based model for post-implantation human amniotic sac development	3D-hE-amnioid	Shao et al., 2017; Zheng et al. (2019)
3D human epiblast model	3D-hE-gastruloid	Simunovic et al. (2019)
Gastruloids, gastruloids	3D-mE-post-gastruloid	Beccar et al. (2018); van den Brink et al., (2014)
Gastruloids, gastruloids	3D-mE-somitoid	van den Brink et al. (2020)
Gastruloids, gastruloids	3D-hE-somitoid	Moris et al. (2020)
Neuroloids	2D-hE-ectotoid	Britton et al., 2019; Haremaki et al. (2019); Xue et al., 2018

m, mouse; EP, embryonic and expanded PSCs; EPS, extended pluripotent stem cells; ET, ETS; ETX, embryonic, trophoblast, and extra-embryonic endoderm stem cells; PSC, pluripotent stem cell; TSC, trophoblast stem cell; XEN, extra-embryonic endoderm stem cells.

Developing nomenclature systems or regulation of naming entities is not novel in science. Genes were also given different names and needed a new system to determine the default name. For instance, the gene BRCA1, the gene associated with breast cancer in humans that encodes a protein associated with DNA repair, has also been known as IRIS, PSCP, BRCAI, BRCC1, FANCS, PNCA4, RNF53, BROVCA1, and PPP1R53. The HUGO Gene Naming Committee (HGNC) was developed to approve gene names and symbols (https://www.genenames.org/about/).

## Conclusions

Naming something has power in science. It helps identify what is being discovered and shapes future discussions about it. Embryoid research is a sensitive area since it is often linked with early human development and the human embryo, with all the complex moral discussions that relate to them. Public understanding of research, its importance, as well as its limitations, is vital for creating an environment accepting of embryoid research.

One key is avoiding names that create confusion and controversy by implying the research can do more than it actually does. We suggest two options for fixing this issue. First, the field should determine a general term to use with the broad public. Determining the most appropriate name should be resolved sooner rather than later, and it needs to be universally utilized in the future, especially when engaging with the public and the media.

Second, a nomenclature structure should be developed to describe specific models. In addition to helping scientists understand each other and new researchers entering the field, a nomenclature system could help policymakers and regulators understand the utility of these entities, their limits, and differentiate between different models. This is especially important in the United States, where federal funding for human embryo research is banned but human ESC research is not (Hyun et al., 2020). The nomenclature structure could be used beyond embryoids and for other types of organoids to help differentiate them from each other.

While we provided specific recommendations, the goal of this paper is not necessarily to push for one specific name or nomenclature structure. Instead, we recommend a more robust discussion and the development of a nomenclature with the community that will use it. For it to be successful, it will require scientists to use the new terms and journals to encourage the scientific community to use them in publications. If done, clear definitions of different models could also be created to help with future public discussions regarding new research.

## Author contributions

All authors contributed equally to this work and are listed in alphabetical order.

## Conflicts of Interest

The authors declare no competing interests.
